# Crystallization and preliminary X-ray diffraction analysis of YidC, a membrane-protein chaperone and insertase from *Bacillus halodurans*

**DOI:** 10.1107/S2053230X14012540

**Published:** 2014-07-23

**Authors:** Kaoru Kumazaki, Tomoya Tsukazaki, Tomohiro Nishizawa, Yoshiki Tanaka, Hideaki E. Kato, Yoshiko Nakada-Nakura, Kunio Hirata, Yoshihiro Mori, Hiroaki Suga, Naoshi Dohmae, Ryuichiro Ishitani, Osamu Nureki

**Affiliations:** a Department of Biological Sciences, Graduate School of Science, The University of Tokyo, 7-3-1 Hongo, Bunkyo-ku, Tokyo 113-0033, Japan; b Global Research Cluster, RIKEN, 2-1 Hirosawa, Wako-shi, Saitama 351-0198, Japan; c Department of Systems Biology, Graduate School of Biological Sciences, Nara Institute of Science and Technology, 8916-5 Takayama-cho, Ikoma-shi, Nara 630-0192, Japan; d JST, PRESTO, 4-1-8 Honcho, Kawaguchi, Saitama 332-0012, Japan; e Department of Molecular and Cellular Physiology, Stanford University School of Medicine, Stanford University, Stanford, CA 94305, USA; f Department of Cell Biology, Graduate School of Medicine, Kyoto University, Yoshidakonoe-cho, Sakyo-ku, Kyoto 606-8501, Japan; g SR Life Science Instrumentation Unit, RIKEN SPring-8 Center, 1-1-1 Kouto, Sayo-cho, Sayo-gun, Hyogo 679-5148, Japan; h Department of Chemistry, Graduate School of Science, The University of Tokyo, 7-3-1 Hongo, Bunkyo-ku, Tokyo 113-0033, Japan

**Keywords:** YidC, membrane-protein insertase, lipidic cubic phase, *Bacillus halodurans*

## Abstract

YidC, a member of the YidC/Oxa1/Alb3 family, inserts proteins into the membrane and facilitates membrane-protein folding in bacteria. YidC plays key roles in both Sec-mediated integration and Sec-independent insertion of membrane proteins. Here, *Bacillus halodurans* YidC2, which has five transmembrane helices conserved among the other family members, was identified as a target protein for structure determination by a fluorescent size-exclusion chromatography analysis. The protein was overexpressed, purified and crystallized in the lipidic cubic phase. The crystals diffracted X-rays to 2.4 Å resolution and belonged to space group *P*2_1_, with unit-cell parameters *a* = 43.9, *b* = 60.6, *c* = 58.9 Å, β = 100.3°. The experimental phases were determined by the multiwavelength anomalous diffraction method using a mercury-derivatized crystal.

## Abstract 2

YidC, a membrane-protein chaperone/insertase from *B. halodurans*, was expressed, purified and crystallized in the lipidic cubic phase. An X-ray diffraction data set was collected to 2.4 Å resolution.

## 1. Introduction   

Correct membrane insertion, folding and assembly of newly synthesized membrane proteins are essential for their functions. The Sec translocon (SecYEG in bacteria and Sec61 in eukaryotes), a protein-conducting channel that is conserved in all three domains of life, mediates the translocation of secretory proteins across the plasma membrane as well as the integration of membrane proteins into the lipid bilayer (Park & Rapoport, 2012 ▶). In bacteria, the functions of YidC in membrane-protein insertion are essential for cell viability (Samuelson *et al.*, 2000 ▶). YidC plays two different roles in membrane-protein insertion (Dalbey *et al.*, 2014 ▶). Firstly, YidC works as a membrane-protein chaperone, which facilitates membrane-protein folding and assembly in cooperation with the Sec translocon. YidC transiently receives the transmembrane (TM) segment released from the Sec translocon (Urbanus *et al.*, 2001 ▶; Sachelaru *et al.*, 2013 ▶), and prevents protein misfolding induced by nonspecific interactions with other TM segments. YidC also interacts with the proton-driven SecDF (Tsukazaki *et al.*, 2011 ▶), which may also participate in this Sec-dependent pathway (Xie *et al.*, 2006 ▶). Secondly, YidC mediates membrane-protein insertion independently. In this Sec-independent pathway, YidC is responsible for the insertion of several single or double membrane-spanning proteins, such as the F_o_ subunit c of the ATP synthase (F_o_c), subunit II of cytochrome o oxidase (CyoA) and the mechanosensitive channel MscL.

YidC is a member of the YidC/Oxa1/Alb3 family and contains the five core TM helices conserved among the other family members: Oxa1 in mitochondria and Alb3 in chloroplasts (Funes *et al.*, 2011 ▶; Saller *et al.*, 2012 ▶). Gram-negative bacteria have one copy of YidC, whereas some species of Gram-positive bacteria possess two YidC paralogues: YidC1 (also known as SpoIIIJ) and YidC2 (also known as YqjG) (Funes *et al.*, 2011 ▶). The YidC proteins from Gram-negative bacteria possess an additional TM helix and a large periplasmic region formed by the N-termini of the core TM region. Structural information about YidC has been limited to the crystal structures of the large periplasmic domain and electron-microscopic studies (Oliver & Paetzel, 2008 ▶; Ravaud *et al.*, 2008 ▶; Kohler *et al.*, 2009 ▶; Seitl *et al.*, 2014 ▶).

## 2. Materials and methods   

### 2.1. Construction   

**Figure 1:**
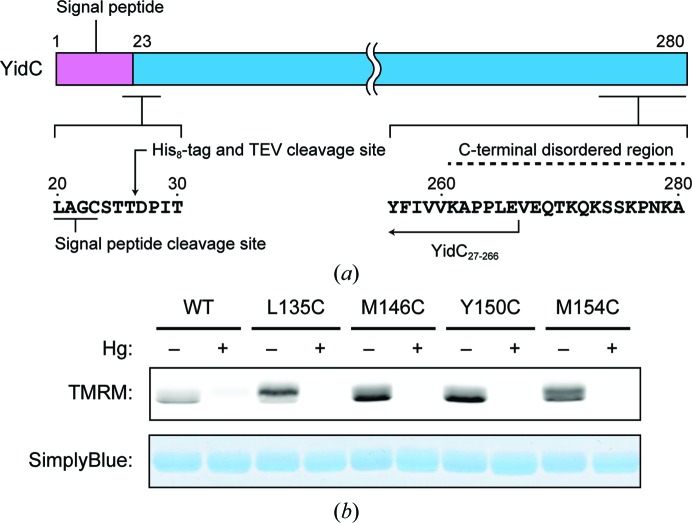
Preparation of YidC_27–266_ and cysteine mutants of YidC_27–266_. (*a*) Construction of YidC_27–266_. The amino-acid sequences of the N- and C-terminal regions of YidC are shown. A His_8_ tag followed by the *Tobacco etch virus* (TEV) protease cleavage site were introduced at the 26th position. The C-terminal 14 residues were removed. (*b*) Methylmercury chloride labelling of wild-type YidC_27–266_ and cysteine mutants of YidC_27–266_. The tetramethylrhodamine-5-maleimide (TMRM) signals (top) and the gel stained with SimplyBlue SafeStain (bottom).

The *yidC* genes from the genomic DNA of 26 thermophilic and halophilic bacteria were cloned into the plasmid pCGFP-BC (Kawate & Gouaux, 2006 ▶), using the *Nco*I or *Eco*RI and *Hin*dIII or *Xho*I sites. The resulting plasmids, encoding C-terminally GFP-His_8_-tagged YidC, were used for a fluorescent size-exclusion chromatography (FSEC) analysis (described in the next section). Further modifications of the plasmid encoding *Bacillus halodurans* YidC2 (YidC) were performed by a PCR-based method, as follows. A His_8_ tag followed by a *Tobacco etch virus* (TEV) protease cleavage site (ENLYFQGQ) was introduced between the 26th and 27th residues of YidC, and the C-terminal 19 and 14 residues followed by the GFP-His_8_ tag were removed from YidC to produce YidC_27–261_ and YidC_27–266_, respectively (Fig. 1 ▶ *a*). For mercury derivatization, cysteine mutations were introduced by site-directed mutagenesis.

### 2.2. Fluorescent size-exclusion chromatography (FSEC)   

FSEC was performed as described previously with modifications (Kawate & Gouaux, 2006 ▶). The C-terminally GFP-His_8_-tagged YidC proteins were overproduced in *Escherichia coli* C41(DE3) or BL21(DE3) cells harbouring pRARE (Novagen) and the pCGFP-BC-based plasmid under a variety of growth conditions by changing key parameters such as culture temperature, duration and induction timing. The cells were grown in 5 ml LB medium supplemented with appropriate antibiotics. The cells were harvested, resuspended in buffer *A* (20 m*M* Tris–HCl pH 8.0, 300 m*M* NaCl, 0.1 m*M* phenylmethylsulfonyl fluoride) and disrupted by sonication with a Bioruptor (Cosmo Bio, UCW-310). After centrifugation at 13 000*g* for 30 min, the supernatant was solubilized with 2% *n*-dodecyl-β-d-maltoside (DDM) in buffer *A*. The insoluble material was removed by ultracentrifugation (Beckman Coulter, TLA55 rotor, 71 680*g*, 30 min) and the supernatant was loaded onto a Superdex 200 10/300 column (GE Healthcare) equilibrated in buffer *B* (20 m*M* Tris–HCl pH 8.0, 300 m*M* NaCl, 0.1% DDM). The fluorescence in the eluate was detected by a fluorometer (Shimadzu, RF-20Axs) with excitation at 480 nm and emission detection at 512 nm.

### 2.3. Expression and purification   

The plasmid encoding YidC was introduced into *E. coli* C41(DE3) cells harbouring pRARE and the proteins were purified as follows. The cells were grown in a 5 l LB culture at 37°C to an *A*_600_ of approximately 0.7 and gene expression was induced with 1 m*M* isopropyl β-d-1-thiogalactopyranoside at 15°C for 16 h. The cells were harvested by centrifugation at 4500*g* for 10 min. The pellet was resuspended in buffer *A* and disrupted by two passages through a Microfluidizer (Microfluidics) at 105 MPa. After centrifugation at 25 000*g* for 30 min, the supernatant was ultracentrifuged (Beckman Coulter, Ti45 rotor, 138 000*g*, 1 h) and the membrane fraction was collected. The membrane fraction was solubilized in buffer *C* [20 m*M* Tris–HCl pH 8.0, 300 m*M* NaCl, 20 m*M* imidazole, 1% DDM, 0.1% cholesteryl hemisuccinate (CHS)]. The insoluble material was removed by ultracentrifugation (138 000*g*, 30 min) and the supernatant was mixed with 5 ml Ni–NTA Superflow resin (Qiagen) in an Econo-Column (Bio-Rad). After binding for 1 h at 4°C, the resin was washed with buffer *D* (20 m*M* Tris–HCl pH 8.0, 300 m*M* NaCl, 20 m*M* imidazole, 0.1% DDM, 0.01% CHS) and YidC was eluted in the same buffer supplemented with 300 m*M* imidazole. The N-terminal residues and the His_8_ tag were cleaved by His-tagged TEV protease (laboratory stock) and the sample was reloaded onto the Ni–NTA column to remove the TEV protease. The flowthrough fraction containing YidC was collected, concentrated and loaded onto a Superdex 200 10/300 column (GE Healthcare) equilibrated in buffer *E* (20 m*M* Tris–HCl pH 8.0, 300 m*M* NaCl, 0.1% DDM, 0.01% CHS). For crystallization, the purified protein was concentrated to 6 mg ml^−1^ with a centrifugal filter device (Millipore, 50 kDa molecular-weight cutoff) and dialyzed against a buffer consisting of 1 m*M* Tris–HCl pH 8.0, 0.05% DDM, 0.005% CHS. Typically, we obtained approximately 1 mg purified YidC from a 5 l culture. The protein concentration was estimated by assuming an *A*_280_ of 1.376 for a 1 mg ml^−1^ solution. For the mercury derivative, the single cysteine mutants were purified by the same procedure used for the purification of the native protein. Before crystallization, the proteins were incubated with 2 m*M* methylmercury chloride at room temperature for 1 h.

### 2.4. Crystallization   

The protein was mixed with monoolein (Nu-Chek Prep) at a 2:3(*w*:*w*) protein-to-lipid ratio using the twin-syringe method (Caffrey, 2009 ▶). Aliquots (50 nl) of the mixture were spotted onto a Lipidic Cubic Phase Screening plate (Swissci) and overlaid with 800 nl precipitant solution using a Mosquito LCP (TTP LabTech). Initial crystallization conditions were searched for using screening kits including MemMeso and MemGold (Molecular Dimensions) and home-made grid-screening kits containing buffers, polyethylene glycols and salts. The initial hits were optimized by manually spotting aliquots (70 nl) onto a glass-slide-based plate and overlaying 1 µl precipitant solution. The mercury-derivative crystals were obtained under conditions similar to those used for the native protein. The crystals grew to full size in 2–3 weeks at 20°C.

### 2.5. Data collection and preliminary crystallographic analysis   

The crystals were flash-cooled using reservoir solution supplemented with 20% polyethylene glycol 500 dimethylether (PEG 500 DME) and 20% glycerol as a cryoprotectant and were stored in liquid nitrogen. All X-ray diffraction experiments were performed on the BL32XU beamline at SPring-8 using an MX225HE detector. A native data set was collected using a microfocused X-ray beam with 1 µm width and 10 µm height at a wavelength of 1 Å (Hirata *et al.*, 2013 ▶). The total oscillation range covered was 180°, with an oscillation range of 1.5° per image. A multiwavelength anomalous diffraction (MAD) data set was collected from a mercury-derivative crystal at wavelengths of 1.00000 Å (the Hg peak) and 1.00945 Å (the Hg inflection). The total oscillation range covered was 360° for all data sets, with an oscillation range of 2.5° per image. Diffraction data were processed using *HKL*-2000 (HKL Research Inc., Otwinowski & Minor, 1997 ▶). The values of CC_1/2_ were calculated with *PHENIX* (Afonine *et al.*, 2012 ▶). One Hg site was identified with *SHELXD* (Sheldrick, 2008 ▶). The initial phases were calculated using *SHARP* (de La Fortelle *et al.*, 1997 ▶), followed by solvent flattening with *SOLOMON* (Abrahams & Leslie, 1996 ▶). The main chain was traced by automated model building using *RESOLVE* (Terwilliger & Berendzen, 1999 ▶). Model building and refinement were performed using *Coot* (Emsley *et al.*, 2010 ▶) and *PHENIX* (Afonine *et al.*, 2012 ▶), respectively.

### 2.6. Fluorescence detection of heavy-atom labelling (FD-HAL)   

FD-HAL was performed as described previously with minor modifications (Chaptal *et al.*, 2010 ▶). The purified proteins, concentrated to 125 µ*M* (∼3 mg ml^−1^), were incubated at room temperature with 1 m*M* methylmercury chloride (from a 100 m*M* stock in dimethyl sulfoxide) for 1 h. An equal volume of buffer *E* supplemented with 250 µ*M* tetramethylrhodamine-5-maleimide (TMRM) was then added and the reaction was incubated for 15 min at room temperature. The reaction was stopped by the addition of an equal volume of SDS–PAGE loading buffer (250 m*M* Tris–HCl pH 6.8, 4% SDS, 20% glycerol, 0.01% bromophenol blue, 3% β-mercaptoethanol). The samples were analyzed by SDS–PAGE and the TMRM signals were detected by a Typhoon FLA 9500 imager (GE Healthcare). Subsequently, the gel was stained with SimplyBlue SafeStain (Life Technologies).

## 3. Results and discussion   

### 3.1. Target screening and purification   

We screened the YidC proteins from 26 thermophilic or halophilic bacteria by FSEC analysis (Kawate & Gouaux, 2006 ▶). This screen identified *B. halodurans* YidC2 (YidC) as a suitable candidate for crystallization because of its high expression and good monodispersity. At first, we tried to purify YidC solubilized by DDM without CHS. However, the YidC in the DDM solution was unstable and aggregated in a few days during purification. To improve the stability, we purified the protein in a detergent solution supplemented with CHS, which was successfully used to stabilize G-protein-coupled receptors (GPCRs) and some mammalian transporters solubilized in detergent solution (Sonoda *et al.*, 2010 ▶). As a result, the stability of YidC in the detergent solution was improved and the protein was successfully purified by a three-step column chromatography procedure. These results suggested that CHS can stabilize bacterial membrane proteins as well as GPCRs, which to our knowledge has not previously been reported.

### 3.2. Crystallization   

**Figure 2:**
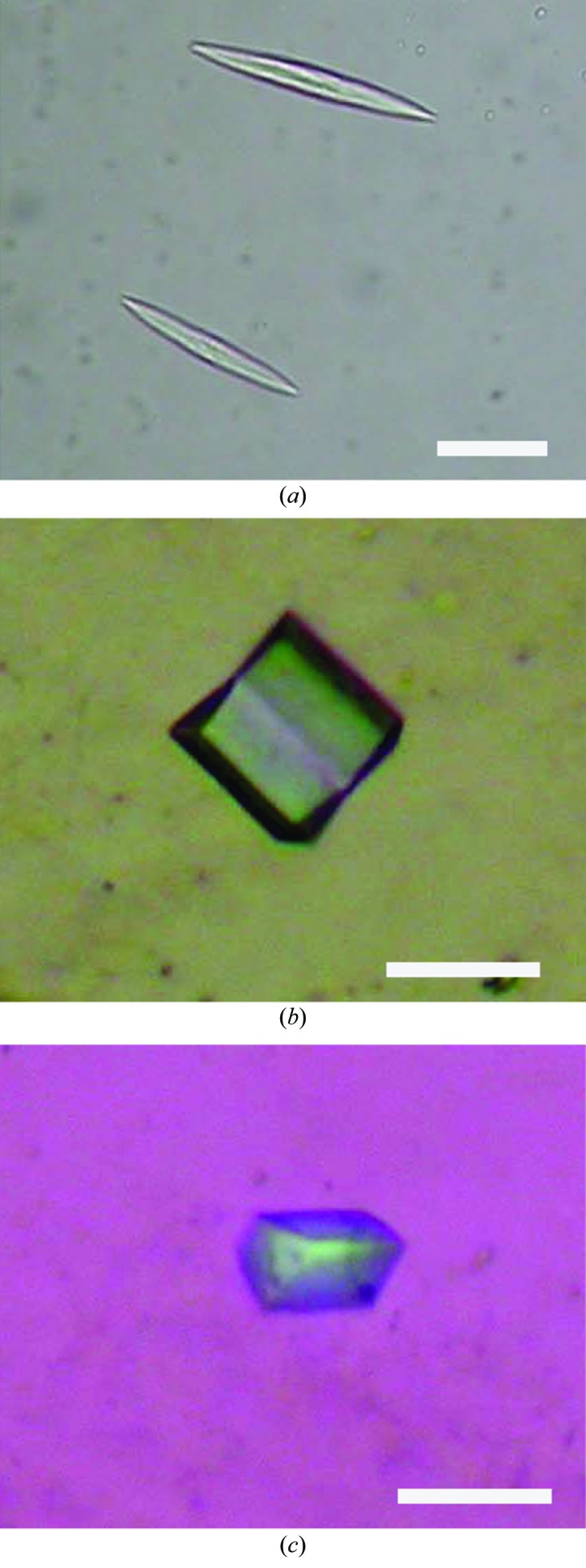
Crystals of YidC. (*a*) Crystals of YidC. (*b*) Crystals of native YidC_27–266_. (*c*) Crystals of mercury-derivatized Y150C YidC_27–266_ mutant. The scale bars represent 30 µm.

The purified protein was crystallized in the lipidic cubic phase. Initially, plate-shaped crystals with approximate dimensions of 80 × 10 × 5 µm were obtained in a reservoir solution consisting of 20% PEG 400, 50 m*M* Na MES pH 6.5, 30 m*M* MgCl_2_, 1 m*M* CdCl_2_ (Fig. 2 ▶ *a*). However, despite optimization of the conditions, the crystals only diffracted to ∼5 Å resolution. YidC has the putative cleavage site of a type II signal peptidase, suggesting that the N-terminus of YidC is lipid-modified after cleavage of the N-terminal signal peptide (Tjalsma *et al.*, 2003 ▶). Post-translational modifications, such as lipidation or glycosylation, often impede the production of high-quality crystals owing to heterogeneity and flexibility. Therefore, to cleave the N-terminal lipid-modified residue, we introduced a His_8_ tag followed by a TEV protease-cleavable sequence between residues 26 and 27 of YidC. In addition, we introduced a stop codon at the 262nd and 267th residue positions of YidC (Fig. 1 ▶ *a*), because the C-terminal residues of YidC were predicted to be disordered by *DISOPRED* (Ward *et al.*, 2004 ▶). We then performed crystallization screening of the truncated constructs, YidC_27–261_ and YidC_27–266_, and obtained cuboid-shaped crystals of YidC_27–266_ with approximate dimensions of 10 × 10 × 5 µm. The crystals were obtained in a reservoir solution consisting of 30% PEG 500 DME, 1 m*M* CdCl_2_, 100 m*M* sodium cacodylate pH 6.0. These initial conditions were optimized by varying the pH, the types and concentrations of salt and the PEG in the reservoir solution. Finally, crystals with approximate dimensions of 30 × 30 × 10 µm were obtained in reservoir solutions consisting of 28–32% PEG 500 DME, 2.5 m*M* CdCl_2_, 100 m*M* sodium cacodylate pH 6.0 (Fig. 2 ▶ *b*).

### 3.3. Data collection and preliminary crystallographic analysis   

**Figure 3:**
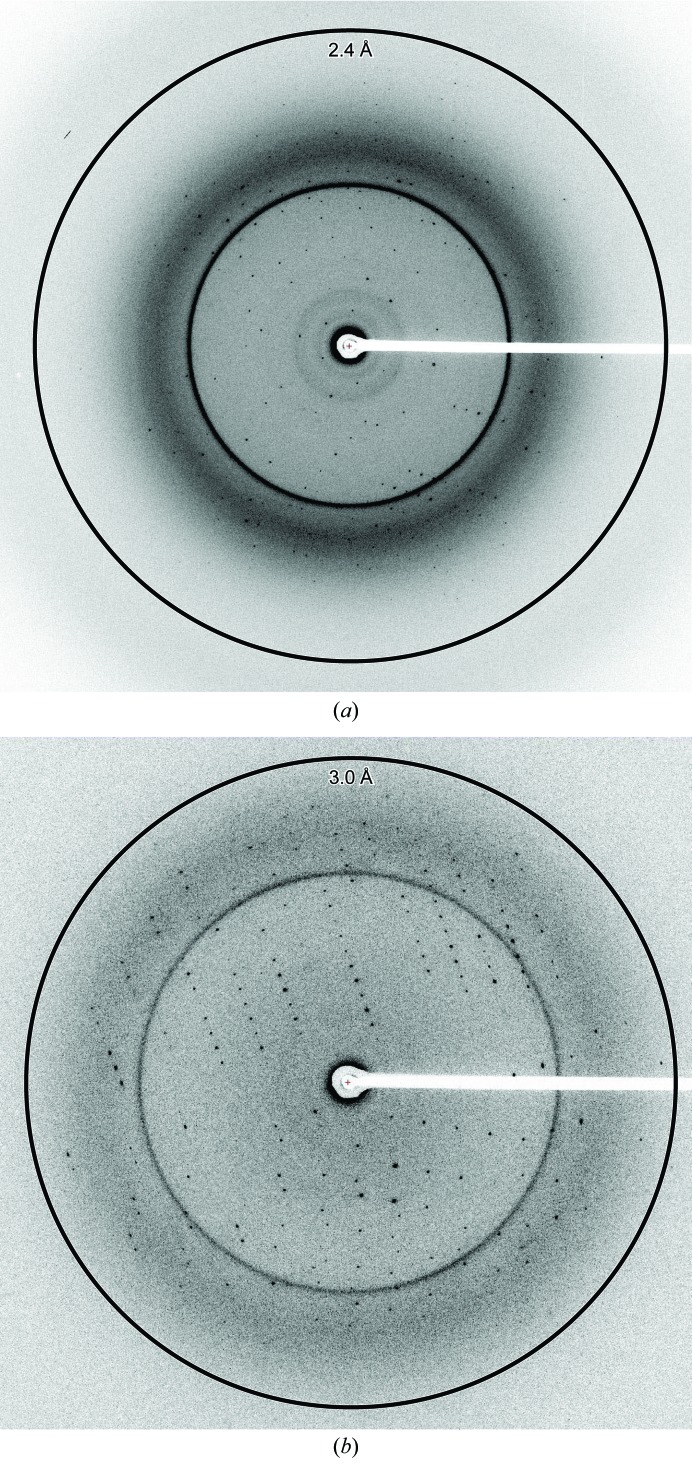
X-ray diffraction pattern of YidC. (*a*) X-ray diffraction pattern of the native crystal of YidC_27–266_. (*b*) X-ray diffraction pattern of the mercury-derivatized crystal of the Y150C YidC_27–266_ mutant. The rings indicate 2.4 Å (*a*) and 3.0 Å (*b*) resolution.

The native crystals diffracted X-rays to 2.4 Å resolution (Fig. 3 ▶ *a*) and belonged to space group *P*2_1_, with unit-cell parameters *a* = 43.9, *b* = 60.6, *c* = 58.9 Å, β = 100.3°. The data-collection statistics are summarized in Table 1 ▶. The calculated Matthews coefficient (*V*_M_) of 2.76 Å^3^ Da^−1^ suggested the presence of one molecule (27.9 kDa) in the asymmetric unit, with a solvent content of 55.5%.

YidC_27–266_ has one cysteine residue (Cys136). For mercury derivatization, we tested whether an Hg atom could access this cysteine, using FD-HAL (Chaptal *et al.*, 2010 ▶). The result showed a weak signal of TMRM, suggesting that Cys136 was not readily accessible to the Hg atom (Fig. 1 ▶ *b*). Therefore, we prepared four cysteine mutants (L135C, M146C, Y150C and M154C) of YidC_27–266_ and tested their binding to Hg atoms by FD-HAL. The results showed stronger TMRM signals of the mutants than the wild-type YidC_27–266_, and the signals were eliminated by methylmercury chloride pre-labelling (Fig. 1 ▶ *b*). This suggested that these mutants can bind mercury atoms more strongly than wild-type YidC_27–266_. The mercury-derivatized crystals of the mutants were obtained under conditions similar to those used for the native protein (Fig. 2 ▶ *c*).

The mercury-derivatized crystal of the Y150C YidC_27–266_ mutant diffracted to 3.0 Å resolution (Fig. 3 ▶ *b*). We collected two-wavelength MAD data sets from the crystal at the absorption peak and the inflection point (Table 1 ▶). We identified one Hg site with *SHELXD* (Sheldrick, 2008 ▶). The initial phase was calculated using *SHARP* (de la Fortelle *et al.*, 1997 ▶), followed by solvent flattening with *SOLOMON* (Abrahams & Leslie, 1996 ▶), which resulted in an interpretable electron-density map. We then built an atomic model of YidC and refined the structure at 2.4 Å resolution to facilitate further investigations of the mechanisms of YidC-mediated membrane-protein insertion (Kumazaki *et al.*, 2014 ▶).

## Figures and Tables

**Table 1:** Data collection and processing Values in parentheses are for the outer shell.

		YidC_27–266_ Y150C (mercury-derivatized)	
	Native YidC_27–266_	Peak	Inflection
Diffraction source	BL32XU, SPring-8	BL32XU, SPring-8	BL32XU, SPring-8
Wavelength (Å)	1.00000	1.00000	1.00945
Temperature (°C)	−173	−173	−173
Detector	RayoniX MX225HE CCD	RayoniX MX225HE CCD	RayoniX MX225HE CCD
Crystal-to-detector distance (mm)	230	300	300
Rotation range per image (°)	1.5	2.5	2.5
Total rotation range (°)	180	360	360
Exposure time per image (s)	1	1	1
Space group	*P*2_1_	*P*2_1_	
*a*, *b*, *c* (Å)	43.9, 60.6, 58.9	43.8, 59.7, 58.6	
β (°)	100.3	100.3	
Mosaicity (°)	0.675	0.673	0.719
Resolution range (Å)	50.00–2.40 (2.44–2.40)	50–3.00 (3.05–3.00)	50–3.00 (3.05–3.00)
Total No. of reflections	28388	35014	32433
No. of unique reflections	10889	6017	6056
Completeness (%)	91.0 (85.6)	99.3 (98.3)	99.2 (98.3)
Multiplicity	2.6 (1.9)	5.8 (4.4)	5.4 (3.5)
〈*I*/σ(*I*)〉	22.4 (2.82)	29.3 (4.72)	21.5 (2.75)
*R*_meas_ (%)	9.8 (50.9)	9.7 (39.5)	11.1 (53.6)
CC_1/2_	0.995 (0.690)	0.997 (0.559)	0.996 (0.114)
Overall *B* factor from Wilson plot (Å^2^)	42.58	63.74	63.08
